# Persistent hypotension and other complications of celiac plexus neurolysis: A case report and literature review

**DOI:** 10.1002/ccr3.7505

**Published:** 2023-06-08

**Authors:** Rhuann Pontes dos Santos Silva, Arthur José Maia Lopes, Roberto Borges Bezerra, Rafael Albanez Andrade, Rebeca Gonelli Andrade, Larissa Monteiro Ferreira da Costa, Carolina Albanez de Albuquerque da Cunha Andrade

**Affiliations:** ^1^ Catholic University of Pernambuco Recife Brazil; ^2^ CERNEDOR, Real Hospital Português Recife Brazil; ^3^ NEOH, Hospital Memorial São Jose Recife Brazil; ^4^ Pain Center IMIP Cernedor, RealNeuro Recife Brazil; ^5^ Division of Rheumatology CERNEDOR, Hospital das Clínicas Recife Brazil

**Keywords:** abdominal pain, adrenal cortex hormones, celiac plexus, postural hypotension

## Abstract

**Key Clinical Message:**

Persistent hypotension is a rare complication of celiac plexus neurolysis. It is important to know what are the main and rare complications and how to treat these in patients who undergo CPN.

**Abstract:**

Celiac plexus neurolysis is an effective treatment for visceral abdominal pain in oncological patients. Although it rarely has complications, some side effects may occur. A patient with visceral abdominal pain who developed long‐lasting orthostatic hypotension and was treated with the use of corticosteroids after a neurolytic celiac plexus block for intractable pain. We describe a rare complication and its treatment and we emphasize the importance of having a guide for the management and treatment of rare complications. We also suggest that every patient be informed about complications, from the most common to the rarest.

## Fluoroscopy shows the positioning of the needles in front of the vertebral body, for injection of absolute alcohol into the celiac plexus ganglia.

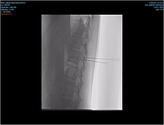

## 1 INTRODUCTION

Abdominal pain is a common debilitating problem in patients with abdominal neoplasms and dramatically affects their quality of life and life expectancy. Pain can be controlled with conventional analgesics, such as acetaminophen, nonsteroidal anti‐inflammatory drugs, and opioids, in majority of oncological patients. However, some patients may experience refractory pain or report adverse effects that prevent their further use. In these patients, interventional pain procedures may be indicated. The commonly indicated procedures are morphine intrathecal delivery, midline myelotomy, and celiac plexus neurolysis (CPN). Intrathecal drug delivery systems are expensive and pose a risk of infection. Patients who undergo midline myelotomy may suffer from bowel and bladder dysfunction and loss of proprioception and vibratory sensation. Neurolysis, however, is a very effective and minimally invasive procedure and is commonly chosen as the initial therapy for the treatment of visceral abdominal pain in cancer patients.

Celiac plexus innervates the liver, pancreas, diaphragm, spleen, stomach, small bowel, ascending and proximal transverse colon, adrenal glands, kidneys, aorta, and mesentery; hence, CPN can be performed to treat upper abdominal pain. Surgical techniques for celiac plexus blockade and neurolysis using both the anterior and posterior approaches to the plexus have been published in the literature. CPN is performed by injecting ethanol into or by radiofrequency ablation of the celiac plexus, leading to permanent disruption of pain transmission.^1^

Although CPN is a low‐risk procedure, some adverse effects have been documented. Most of the effects are transient, and serious complications occur only in less than 2% of cases. The most common adverse effects are diarrhea, hypotension, constipation, nausea and vomiting, pain, and neurological adverse events. Some symptoms are less common after CPN, such as persistent hypotension.

We describe the case of a patient with intractable, visceral abdominal pain who developed long‐lasting postural hypotension after a neurolytic celiac plexus block. This article aims to review the main complications involved in the surgical technique of CPN and discuss the treatment of each of these complications.

## 2 CASE REPORT

A 41‐year‐old woman diagnosed with breast cancer received neoadjuvant chemotherapy (doxorubicin + cyclophosphamide + paclitaxel) and underwent right breast radical mastectomy. Despite the treatment, the patient developed brain metastases, which were treated by radiosurgery, and lymph‐node metastases (mediastinal, pancreatic, mesenteric, and retroperitoneal). Subsequently, the patient developed deep, constant upper abdominal pain. The pain was not adequately relieved even with the prescription of dyprione, pregabalin, and opioids such as morphine, methadone, and oxycodone. The oncology team decided to perform CPN for the treatment of her refractory abdominal pain.

**FIGURE 1:**
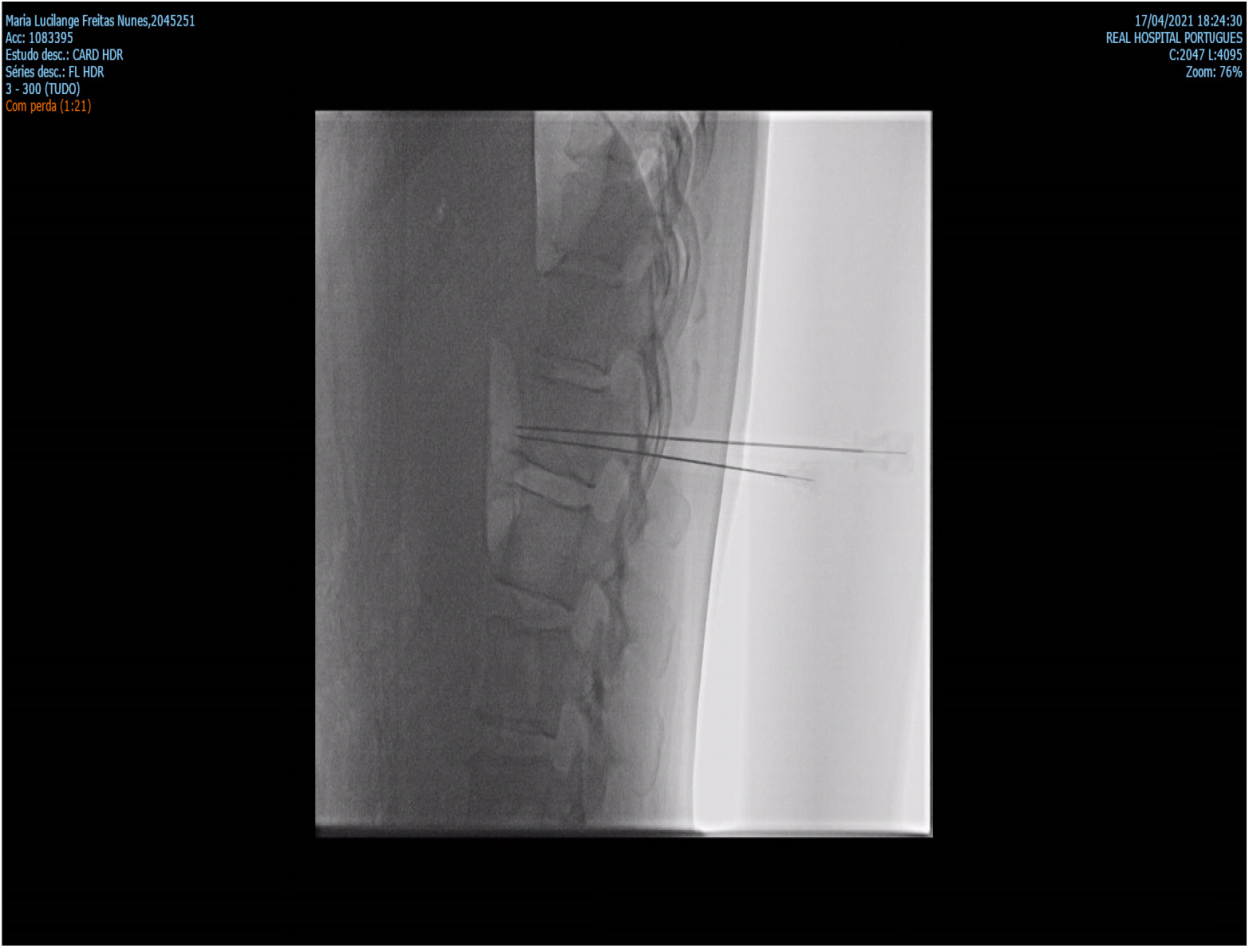
Fluoroscopy shows the positioning of the needles in front of the vertebral body, for injection of absolute alcohol into the celiac plexus ganglia.

CPN was performed under general anesthesia by injecting 60 mL of absolute alcohol into the celiac plexus ganglia, guided by fluoroscopy (Figure 1). There were no complications, and the patient was discharged 24 h after the procedure. She reported to be pain‐free at the time of discharge. One week after CPN, the patient complained of abdominal pain, which was controlled with the use of oxycodone 20 mg BID. The patient also developed episodes of dizziness, fainting, and blurred vision on standing upright. Blood pressure measurements in the standing position showed sustained hypotension. The patient was admitted to receive intravenous hydration and a high‐sodium diet; however, there was no improvement in her blood pressure levels. Thus, we started her on fludrocortisone; she achieved and maintained normal blood pressure levels at a dose of 0.2 mg per day. Thereafter, the patient reported an improvement in her quality of life. The patient received cisplatin and gemcitabine as adjuvant chemotherapy. Despite the oncological treatment, there was disease progression, and the patient died 3 months after the procedure.

## 3 DISCUSSION

Persistent hypotension is a rare complication of celiac plexus neurolysis. In this case, the hypotension was refractory to intravenous hydration and a sodium‐rich diet and improved only with the use of corticosteroids. Although the occurrence of transient post‐procedural hypotension has been well documented in the literature, chronic postural hypotension after CPN remains a rare and poorly documented entity.

Yousefshahi and Tahmasebi (2018)^2^ described a case of persistent hypotension after CPN in which blood pressure was restored with corticosteroid intake, thereby demonstrating the potential of corticosteroids in its treatment. Corticosteroids maintain or improve blood pressure by increasing vascular tone and integrity, increasing myocardial contractility and function, increasing vascular responsiveness to catecholamines and angiotensin II, and decreasing capillary leak and maintaining intravascular volume. The celiac plexus contains mainly sympathetic fibers; therefore, the occurrence of persistent postural hypotension is probably related to the loss of sympathetic innervation and consequent splanchnic vasodilation.^3^

The reported patient also experienced an initial worsening of the abdominal pain. This event is not uncommon after CPN and usually resolves spontaneously.

Neurological complications such as paraplegia, paraparesis, sensory deficits, and paresthesia are rare symptoms that occur in only 1% of cases. The probable causes of neurological deficits are direct irritation of the lumbar nerve roots by alcohol and spasms of radicular lumbar arteries, the anterior spinal artery, or the artery of Adamkiewicz. Permanent paraplegia has been reported in only 0.15% of cases, and the attributed cause was ischemia of the spinal cord secondary to damage or spasm of the artery of Adamkiewicz.^4^

Physiotherapy plays an important role in the treatment of patients with paralytic episodes during the procedure and in those with postoperative paraplegia or paresthesia. As the vast majority of cases occur due to lumbar root irritation or temporary arterial spasm, physiotherapy can help restore lower limb function.

Vascular complications are rare after CPN. Anterior transaortic CPN is performed in some hospitals but should be avoided in patients with mural calcification or aortic aneurysms. Kaplan et al.^5^ reported a case of aortic dissection that occurred following a celiac plexus block via the transaortic approach, which led to liver and bowel infarction and eventually, the patient's death. Venous complications have also been known to occur. The treatment for this complication may be medical or surgical. Thrombolytic, anticoagulant, antiplatelet, and antispasmodic agents can be used for its medical management.

Another rare complication of CPN is alcohol intoxication. The intravenous hydration and elevation of the lower limbs apparent to be an effective treatment for acute alcohol intoxication. Many authors had described patients with signs of alcohol intoxication and who developed acetaldehyde syndrome‐like reactions. All patients recovered within a few days with no lasting effects.

Although post‐procedural diarrhea is quite common, severe and prolonged diarrhea rarely occurs. Persistent diarrhea can be life‐threatening if it is not recognized early and treated. Diarrhea can be treated using somatostatin analogs, such as ocreotide. Ocreotide is effective in controlling diarrhea, with its dosage titrated to effect (0.05–0.2 mg daily over a period of 1 week). The cessation of ocreotide therapy may cause the symptoms to reappear.

## 4 CONCLUSION

The main limitation of this study is that it is a single case study. However, it was important to draw attention to this rare complication that may occur after CPN and discuss the most appropriate treatment modalities. This paper was also designed to serve as a guide in the management of complications in patients who undergo CPN. We recommend that every patient undergoing a procedure should be informed about all its complications, and we recommend that all authors report complications so that we can know the actual number of complications related to the procedure.

## AUTHOR CONTRIBUTIONS

**Rhuann Pontes dos Santos Silva:** Conceptualization; data curation; software; writing – original draft; writing – review and editing. **Arthur José Maia Lopes:** Data curation; formal analysis; writing – review and editing. **Roberto Borges Bezerra:** Formal analysis; funding acquisition; investigation. **Rafael Albanez Andrade:** Investigation; methodology; project administration; validation. **Rebeca Gonelli Andrade:** Formal analysis; investigation; software; visualization; writing – original draft; writing – review and editing. **Larissa Monteiro Ferreira da Costa:** Investigation; methodology; project administration; visualization; writing – original draft. **Carolina Albanez de Albuquerque da Cunha Andrade:** Methodology; software; supervision; writing – original draft; writing – review and editing.

## FUNDING INFORMATION

Not applicable.

## CONSENT

Written informed consent was obtained from the patient to publish this report in accordance with the journal's patient consent policy.

## Data Availability

Not applicable.
